# Supplementary material for: PIE-1 SUMOylation promotes germline fates and piRNA-dependent silencing in C. elegans
Source: eLife. 2021 May 18;10:e63300. doi: 10.7554/eLife.63300 (PMC8131105; doi:10.7554/eLife.63300)
Supplement: Supplementary file 5. [file elife-63300-supp5.docx]

**Supplementary File 5**. Strains and alleles used in this study.

| Strain name: Genotype | Source | Method |
| --- | --- | --- |
| *C. elegans*: Strain JJ532: *pie-1(zu154) unc-25(e156)/qc-1* | *Caenorhabditis* Genetics Center | N/A |
| *C. elegans*: Strain OD56: *unc-119(ed3)*III; Itls37[(pAA64)*pie-1p::mCherry::his-58*+ *unc-119(+)*]IV | (McNally et al., 2016) | MosSCI |
| *C. elegans*: Strain CA1199: *unc-119(ed3)*III; ieSi38[*sun-1p::TIR1::mRuby::sun-1 3’UTR, cb-unc-119(+)*]IV | (Zhang et al., 2015) | MosSCI |
| *C. elegans*: Strain WM330: *pie-1(ne4301*[PIE-1::GFP]*)*III | (Kim et al., 2014) | CRISPR |
| *C. elegans*: Strain WM331: *pie-1(ne4302*[PIE-1::3xFLAG]*)*III | (Kim et al., 2014) | CRISPR |
| *C. elegans*: Strain WM332: *pie-1(ne4303*[PIE-1(K68R)]*)*III | (Kim et al., 2014) | CRISPR |
| *C. elegans*: Strain WM333: *pie-1(ne4304*[PIE-1(K68R)::3xFLAG]*)*III | This study | CRISPR |
| *C. elegans*: Strain WM338: *smo-1(ne4311*[6xHIS::SMO-1]*)*I | This study | CRISPR |
| *C. elegans*: Strain WM650: *pie-1(ne4301*[PIE-1::GFP]*)*III; Itls37[(pAA64)*pie-1p::mCherry::his-58*+ *unc-119(+)*]IV | This study | Cross |
| *C. elegans*: Strain WM651: *ubc-9(ne4446*[UBC-9(G56R)]*)*IV | This study | CRISPR |
| *C. elegans*: Strain WM653: neSi22 [*oma-1::gfp (RNAa), cb-unc-119(+)*] II; neSi10 [*gfp::csr-1(RNAe), cb-unc-119(+)*] IV | (Seth et al., 2018) | Cross |
| *C. elegans*: Strain WM667: *smo-1(ne4346*[10xHIS::SMO-1]*)*I | This study | CRISPR |
| *C. elegans*: Strain WM671: *mep-1(ne4380*[MEP-1::GFP::TEV::3xFLAG]*)*IV | This study | CRISPR |
| *C.elegans*: Strain WM709: *pie-1(ne4443*[PIE-1::Degron::GFP]*), unc-119(ed3)*III; ieSi38[*sun-1p::TIR1::mRuby::sun-1 3’UTR, cb-unc-119(+)*]IV | This study | CRISPR |
| *C. elegans:*Strain WM710: *gei-17(ne4822*[*gei-17 +3nt*, nonsense mutation]*)* I ; neSi22 II; neSi10 IV | This study | CRISPR |
| *C. elegans:*Strain WM711: *gei-17(ne4823*[*gei-17 △4nt*, nonsense mutation]*)* I ;neSi22 II; neSi10 IV | This study | CRISPR |
| *C. elegans:*Strain WM713: neSi22 II; *pie-1(ne4303*[PIE-1(K68R)]*)* III; neSi10 IV | This study | Cross |
| *C. elegans*: Strain WM716: *pie-1(zu154) unc-25(e156)/qc-1*III*; ubc-9(ne4446*[UBC-9(G56R)]*)*IV | This study | Cross |
| *C. elegans*: Strain WM717: *smo-1(ne4346*[10xHIS::SMO-1]*)*I; *pie-1(ne4302*[PIE-1::3xFLAG]*)*III | This study | Cross |
| *C. elegans*: Strain WM718: *smo-1(ne4346*[10xHIS::SMO-1]*)*I; *pie-1(ne4304*[PIE-1(K68R)::3xFLAG]*)*III | This study | Cross |
| *C. elegans*: Strain WM719: *pie-1(ne4302*[PIE-1::3xFLAG]*)*III; *mep-1(ne4380*[MEP-1::GFP::TEV::3xFLAG]*)*IV | This study | Cross |
| *C. elegans*: Strain WM720: *pie-1(ne4304*[PIE-1(K68R)::3xFLAG]*)*III; *mep-1(ne4380*[MEP-1::GFP::TEV::3xFLAG]*)*IV | This study | Cross |
| *C. elegans*: Strain WM721: *smo-1(ne4346*[10xHIS::SMO-1]*)*I; *mep-1(ne4380*[MEP-1::GFP::TEV::3xFLAG]*)*IV | This study | Cross |
| *C. elegans*: Strain WM722: *smo-1(ne4346*[10xHIS::SMO-1]*)*I; *pie-1(ne4303*[PIE-1(K68R)]*)*III*; mep-1(ne4380*[MEP-1::GFP::TEV::3xFLAG]*)*IV | This study | Cross |
| *C. elegans:*Strain WM739: neSi22 II; *pie-1(ne4837*[*pie-1* *△216nt*]*)/+* III; neSi10 IV | This study | CRISPR |
| *C. elegans:*Strain WM740: neSi22 II; *pie-1(ne4838*[*pie-1* *△208nt and 16nt insertion*]*)/+* III; neSi10 IV | This study | CRISPR |
| *C. elegans*: Strain WM741: *pie-1(ne4377*[PIE-1(K68R)::GFP]*)*III | This study | CRISPR |
| *C. elegans:*Strain WM742: *rde-3(ne3370)* I ;neSi22 II; neSi10 IV | This study | CRISPR |
