# Supplementary material for: PIE-1 SUMOylation promotes germline fates and piRNA-dependent silencing in C. elegans
Source: eLife. 2021 May 18;10:e63300. doi: 10.7554/eLife.63300 (PMC8131105; doi:10.7554/eLife.63300)
Supplement: Supplementary file 6. [file elife-63300-supp6.docx]

**Supplementary File 6.** sgRNA sequences and ssOligo donor sequence for CRISPR.

| Target gene/ gRNA sequence | Source |
| --- | --- |
| *pie-1* sgRNA (for c-terminal tagging)  5’-GCTGAGAGAAGAATCCATCG-3’ | (Kim et al., 2014) |
| *smo-1* sgRNA (for N-terminal tagging)  5’-GAGACTCCCGCTATAAACGA-3’ | This study |
| *ubc-9* sgRNA (for G56R)  5’-GGCTCGAACTTGCACTTTGG-3’ | This study |
| *mep-1* sgRNA (for C-terminal tagging)  5’-GCGCAAAAGAAGGAAGACGG-3’ | This study |
| *pie-1 crRNA#1 and crRNA#*2 (for pie-1 indel mutations)  crRNA#1: 5’- atcaaattttcttttccagA-3’  crRNA#2: 5’-ATCTTGAGCGCTTCACGCTT-3’ | This Study |
| *gei-17* crRNA#1 and crRNA#2 (for gei-17 indel mutations)  crRNA#1: 5’-GGTATTTGCCATTGATTATT-3’  crRNA#2: 5’-atATGTTACCGAATAATCAA-3’ | This Study |
| *rde-3* crRNA#1 and crRNA#2 (for ne3370 allele)  crRNA#1(oYD509):5’-TGCAATTCATATCCCACAAG-3’  crRNA#2(oYD510):5’-TGTTTTACGATTGCACATAA-3’ | This Study |
| ssOligo donor sequence | Source |
| rde-3_ne3370_donor  GGCCACTAAAAACAGTGATCTAGACGTTGCAATTCATATCGCAATCGTAAAACAATGGGCAGCCAGTACAAAAGTTAAAG | This study |
